# TaERF109: A Novel ERF Transcription Factor Contributing to Enhanced Resistance to *Puccinia graminis* f. sp. *tritici* Infection in Wheat

**DOI:** 10.3390/pathogens15040387

**Published:** 2026-04-04

**Authors:** Binbin Si, Jiahui Lei, Wufen Zhang, Rong Ma, Yuanyin Cao

**Affiliations:** 1 School of Biological Science and Engineering, North Minzu University, Yinchuan 750021, China; leijiahui0908@163.com (J.L.); zhangwufen1992@163.com (W.Z.); marong1023@163.com (R.M.); 2 Innovation Team for Genetic Improvement of Economic Forest, North Minzu University, Yinchuan 750021, China; 3 College of Plant Protection, Shenyang Agricultural University, Shenyang 110866, China; caoyy6655@163.com

**Keywords:** wheat, *Puccinia graminis* f. sp. *tritici*, *TaERF109*, wheat resistance, VIGS, overexpression

## Abstract

*Puccinia graminis* f. sp. *tritici* (*Pgt*) is responsible for stem rust in wheat, a disease with worldwide occurrence. Ethylene response factors (ERFs), a group of transcription factors (TFs) responsive to ethylene, are essential for managing stress signaling under biotic and abiotic challenges. However, our understanding of ERF TFs’ function in wheat (*Triticum aestivum* L.) resistance against the obligate biotrophic *Puccinia graminis* f. sp. *tritici* remains limited. In this work, we report our findings of the *TaERF109* gene, which is transcriptionally up-regulated by ethylene or *Pgt* infection. TaERF109 is localized in the nucleus of rice protoplasts. Results obtained using the yeast one-hybrid (Y1H) assay support the conclusion that TaERF109 interacts with the AGCCGCC sequence (GCC-box). Transient knockdown of TaERF109 via virus-induced gene silencing (VIGS) increased wheat susceptibility to *Pgt*, accompanied by the down-regulation of three pathogenesis-related (PR) genes, *TaPR1*, *TaPR2*, and *TaPR10*, as confirmed via real-time quantitative PCR. In contrast, the *Agrobacterium*-mediated overexpression of *TaERF109* potentiated resistance of transgenic wheat against *Pgt*. Overall, these results expand the current understanding of the TaERF109 gene’s function in wheat resistance to *Pgt*.

## 1. Introduction

The occurrence of wheat stem rust, attributed to *Puccinia graminis* f. sp. *tritici* (*Pgt*), extends throughout the main wheat-cultivating regions globally [1]. *Pgt* is an obligate parasitic fungus, and it can form the haustorium, an important infection structure, acquiring energy to obtain nutrients from the host during infection [2]. However, the molecular function of ERF in response to wheat stem rust (*Puccinia graminis* f. sp. *tritici*) remains unknown in wheat.

Plants respond to genetic instructions and environmental signals throughout their development. Transcription factors (TFs), which generally harbor conserved DNA-binding motifs, are fundamental to this regulation in higher plants [3]. As special proteins, TFs attach to particular DNA motifs and influence the transcriptional process, directing the transfer of genetic codes from DNA to mRNA [4]. Among these, the APETALA2/ethylene-responsive element binding factor (AP2/ERF) superfamily represents an important subgroup that governs responses to environmental and pathogen-induced stresses [5,6]. Evidence suggests that ERF proteins regulate various pathogenesis-related (PR) genes by binding to the GCC box in their promoters [7]. Growing evidence indicates that AP2/ERFs play a crucial role in plant defense mechanisms against different pathogens. For instance, up-regulating NtERF5 enhances the expression of pathogenesis-related (PR) genes, leading to enhanced resistance to tobacco mosaic virus in tobacco (*Nicotiana tobacum*). Similarly, the overexpression of *Pti4/5/6* boosts resistance against *Erysiphe orontii* and *Pseudomonas syringae* pv *tomato* in Arabidopsis [8]. StERF94 overexpression in potatoes increased the expression of defense-related genes, such as those coding for PR proteins, resulting in enhanced protection against disease spread and decreased fungal growth in plant tissues [9]. Rice MAP kinase BWMK1 phosphorylates OsEREBP1, enhancing its binding to the GCC-box in PR gene promoters, leading to increased PR expression and disease resistance [10].

The AP2/ERF gene superfamily has been systematically analyzed at the genome level in species like *Arabidopsis thaliana* [11], *Brassica napus* [12], *Ananas comosus* [13], *Ipomoea batatas* [14], *Capsicum annuum* [15], *Carya cathayensis* [16], *Fagopyrum tataricum* [17], and *Cucurbita moschata* [18]. Based on domain structure and sequence similarity, this superfamily is commonly categorized into four major families: dehydration-responsive element-binding proteins (DREB), AP2, ERF, and proteins related to ABI3/VP (RAV). AP2 family members carry two AP2 domains, ERFs are limited to one AP2 domain, and RAVs have both AP2 and B3 domains [19].

In wheat, 322 *Ta*AP2/ERF genes have been annotated and systematically categorized into 12 subfamilies distributed across two chromosomes [20]. Within the ERF subfamily, the structural conformation of the AP2 domain’s β-sheet is defined by alanine (position 14) and aspartic acid (position 19)—two characteristically conserved amino acid residues [21]. Within the wheat genome, numerous ERF genes are enmeshed in stress responses. For example, *TaERF8* regulates wheat growth and development [22]. *TaERF1* may be elicited by diverse external cues, including drought, high salt levels, cold, external hormones (e.g., ethylene and salicylic acid), and plant pathogenic fungi (e.g., wheat powdery mildew). *TaERF3* in wheat exhibits pronounced resistance to salt and drought stresses [23]. TaAP2-15, as a transcription factor, positively regulates wheat stripe rust resistance [24].

Although the pivotal functions of AP2/ERF TFs in stress responses are well documented, their specific involvement in the wheat–*Pgt* interaction constitutes a largely uncharted area of research. In the present study, a new ethylene response factor, TaERF109, was identified based on transcriptome data from different development stages of interaction between wheat and *Pgt* in our experiments (data not published). Furthermore, the pivotal role of *TaERF109* in the defensive response of wheat against *Pgt* was analyzed. We concluded that *TaERF109* up-regulates the resistance of wheat to *Pgt*. These observations allow for deeper comprehension of how the ERF subfamily functions in the wheat–*Pgt* pathosystem.

## 2. Materials and Methods

### 2.1. *Plant and Fungal Material*

The present work employed the wheat (*T. aestivum* L.) cultivar ‘Fielder’ and *Pgt* isolate 34C3RTGQM, which demonstrated a compatible interaction. The American soft white wheat cultivar ‘Fielder’, a pastry-type wheat, was released in 1974. It is recognized for its suitability for *Agrobacterium tumefaciens*-mediated transformation and genome editing [25]. Due to the genotype-specific nature of genetic transformation in wheat, ‘Fielder’ stands out as a dependable haplotype for methods utilizing *Agrobacterium tumefaciens*-mediated transformation [25,26,27]. ‘Fielder’ seedlings were cultivated in sterilized soil in 12 cm pots (6 pots) within a growth chamber at 22 °C under 50% relative humidity and a 16 h photoperiod for one week, when they reached the two-leaf seedling stage. The physiologically *Pgt* race 34C3RTGQM was identified and provided by the Institute of Plant Immunity, Shenyang Agricultural University. Subsequently, the seedlings were inoculated with 34C3RTGQM according to a previously described protocol. Initially, a 0.05% Tween-20 solution was sprayed on the leaves with a handheld atomizer to create a water film. Subsequently, fresh urediniospores (1 g) and dried talc, mixed in a ratio of 1:20 (*w*/*w*), were inoculated on the seedlings [28]. To extract RNA, leaf tissue from *Pgt*-inoculated wheat plants was harvested at 5 time points (1, 3, 6, 9, and 12 d), underwent rapid cryopreservation in liquid nitrogen, and subsequently maintained at −80 °C, with tissue samples sprayed with 0.05% Tween-20 acting as the control.

### 2.2. Exogenous Ethylene Treatment

Two-leaf stage (one leaf and one sprout) aboveground were sprayed with 100 μM of ethephon (C_2_H_6_ClO_3_P, Macklin, Shanghai Macklin Biochemical Technology Co., Ltd., Shanghai, China) solution and bagged, and random samplings of six wheat leaves were collected at 0, 3, 6, 9, 12, and 24 h. Three leaves from each sampling stage underwent inoculation with *Pgt* and incubation in an artificial climate chamber, while the remaining three leaves immediately underwent liquid nitrogen cryopreservation and were subsequently maintained at −80 °C for RNA isolation. The *TaERF109* content was detected *via* quantitative real-time fluorescence PCR. The control samples were *T. aestivum* L. leaves sprayed with 0.05% (*v*/*v*) Tween-20 only. Each biological sample was replicated three times.

### 2.3. RNA Isolation, cDNA Preparation, and qRT-PCR

Total RNA isolation was accomplished utilizing the plant RNA extraction kit as per the supplier’s prescribed protocol (Omega Bio-Tek, GA, USA). The RNA samples were converted to cDNA with the RT reagent Kit (PrimeScript™ RT reagent Kit with gDNA Eraser, Takara, Dalian, China). The qRT-PCR assay was performed using TB Green^®^ Premix Ex Taq™ II (Takara, Dalian, China). Primers specific to TaERF109 (GenBank: PX904030) were designed using Primer5 software. In the following amplification process, the primer pair *TaERF109*-F and *TaERF109*-R (Supplementary Table S1) was employed, with the wheat gene *TaGAPDH* [29] used as a normalization control. Quantification of relative expression was performed following the 2^−ΔΔCt^ method [30]. The qRT-PCR data included results from three biological replicates, each group with three technical repetitions.

### 2.4. Cloning of TaERF109 and Sequence Analysis

In order to explore the biological function of the *TaERF109* gene, a clone was made. Primers were designed using Primer5 with reference to the *TaERF109* coding sequence (CDS) [31]. The PCR products were subcloned into the pMD 18-T vector (Takara, Dalian, China) and sequenced [32].

Multiple sequence alignments were carried out using DNAMAN 8.0 (Lynnon Biosoft, California, USA). Identification of the open reading frame (ORF) relied on ORF Finder (http://www.ncbi.nlm.nih.gov/projects/gorf; accessed on 12 November 2025). Phyre2 (http://www.sbg.bio.ic.ac.uk/phyre2/html/page.cgi? id = index; accessed on 12 November 2025) was used for tertiary structure determination.

### 2.5. Subcellular Localization of the TaERF109 Protein

To ascertain TaERF109’s subcellular localization, a rice protoplast transient localization protocol was used [33]. The resulting amplicon underwent purification utilizing the MiniBEST Agarose Gel DNA Extraction Kit Ver. 4.0 (Takara, Dalian, China) and insertion into the PEGOE35S vector using *Bsa*I-HF (NEB, Massachusetts, USA) for digestion and T4 DNA ligase ligation (NEB, Massachusetts, USA). The resulting recombinant plasmid underwent transformation, extraction, and sequencing using the sequencing primer 35S-F/eGFP-cx [34]. The PEGOE35S-BERF109 vector was constructed successfully if the alignment results confirmed that the sequencing results were consistent with the target fragment sequences. Upon plasmid transformation into rice protoplasts [35], green fluorescent protein (GFP) fluorescence was recorded under a Zeiss LSM 700 (Carl Zeiss Microscopy GmbH, Jena, Germany) confocal fluorescence microscope.

### 2.6. BSMV-Mediated TaERF109 Gene Silencing and Evaluation of Plant Disease Resistance

Using the barley stripe mosaic virus (BSMV) system, virus-induced gene silencing (VIGS) was applied to suppress TaERF109 expression [36]. A custom-designed cDNA segment of TaERF109 (Supplementary Table S1) was evaluated for virus-induced gene silencing (VIGS). This segment was generated via reverse transcription PCR utilizing the ApaI restriction site and subsequently inserted into BSMV: γ to modify the native barley stripe mosaic virus (BSMV), yielding BSMV: TaERF109-1 [37]. The silencing constructs, BSMV-γ: TaERF109, were generated by cloning one TaERF109 cDNA fragment into the BSMV-γ vector. The sequence specificity of each insert was validated with BLAST 2.17.0 (http://blast.ncbi.nlm.nih.gov/Blast.cgi). Wheat seedlings were inoculated on their second leaves with BSMV-γ: *TaERF109* [38] and then kept in darkness at 22 ± 2 °C and 50% humidity for 24 h. At day twelve, wheat leaves were inoculated with fresh urediniospores of *Pgt* 34C3RTGQM, followed by leaf sampling at 0, 1, 2, 3, 6, and 12 days post-inoculation (dpi) for histological assay and RNA isolation. TaERF109 relative expression and TaPR1 [1], TaPR2 [1], and TaPR10 [39] gene expression levels were assessed using qRT-PCR. Wheat phenotypes following *Pgt* inoculation were evaluated based on photographs taken at 12 dpi.

### 2.7. Overexpression of TaERF109 and Evaluation of Plant Disease Resistance

PCR amplification of *TaERF109* was implemented using *TaERF109*-TF and *TaERF109*-TR primers (Supplementary Table S1) to yield its full-length CDS. Transgenic wheat plants of the T2 generation were infected with *Pgt*. Symptoms on infected leaves were observed using a camera at 12 dpi, and the lesion area was measured using Photoshop CS5 [40]. The overexpression vector was constructed in the same manner as the overexpression vector for subcellular localization, with the *bar* gene used as a selectable marker. The freeze–thaw approach was employed to introduce the recombinant construct pEGOE35S-BERF109 into *Agrobacterium tumefaciens*. The wheat cultivar ‘Fielder’ provided cotyledonary nodes as explants for *Agrobacterium*-mediated transformation. Infected leaves were incubated in the dark for 24 h with Agrobacterium. Callus growth was induced on a medium supplemented with 50 mg/L of kanamycin B and 400 mg/L of cephalosporin. After 30 to 45 days, the callus was transferred to a medium designed for screening and differentiation to promote bud formation. The resulting buds were then cultured in Murashige and Skoog medium to enhance root development. Once adequately rooted, the seedlings were transplanted into soil in a greenhouse. To clarify the function of *TaERF109* overexpression, the second leaf underwent inoculation with urediniospores of *Pgt* 34C3RTGQM, and the infection results were observed. Positive transgenic wheats were screened using PCR and RT-PCR with specific primers. The disease resistance of *TaERF109*-overexpression (*TaERF109*-OE) wheat was quantified via qRT-PCR. In addition, the *Pgt* resistance of transgenic wheat was examined using an artificial inoculation method on live leaves of TaERF109-overexpressing lines, with the wild-type ‘Fielder’ (WT) used as a comparison. Pathological manifestations on inoculated foliage were documented utilizing a Canon D6000 camera at 12 dpi. Finally, TaERF109 mRNA accumulation in *TaERF109*-OE lines was determined via qRT-PCR.

### 2.8. Determination of Enzyme Activity and Pathogenesis-Related (PR) Genes in TaERF109-Silenced and TaERF109-OE Wheat Seedlings

Leaf material (0.1 g) was ground in 1 mL of phosphate-buffered saline (50 mM, pH 7.8) at low temperature. Centrifugation (12,000 *g*, 20 min, 4 °C) yielded supernatants that were subsequently examined for peroxidase (POD) and superoxide dismutase (SOD) activities as per the procedure delineated by Li et al. [41].

qRT-PCR was used to perform an expression assessment of PR genes (Supplementary Table S1) and the TaERF109 silencing degree in *TaERF109*-silenced plants, with BSMV: γ plants used as the control.

The abundance of PR-related transcripts in *TaERF109*-OE plants was quantified via qRT-PCR, with LC used as the control.

### 2.9. Yeast One-Hybrid (Y1H) Assay

To elucidate the pathogen’s mechanism of resistance associated with TaERF109, the interaction between pHIS2-GCC-box and pGADT7-ERF109 was verified with a Y1H assay using the vector pHIS2-GCC-box constructed with the GCC-box sequence as bait and the vector pGADT7-ERF109 constructed with the *TaERF109* sequence as prey.

PCR-amplified *TaERF109* CDS was subcloned into the pGADT7 vector, and then the Y1H assay was conducted as per the PT3024-1/Yeast Protocols Handbook using the Y187-pHis2 Yeast One-Hybrid Interaction Proving Kit (Coolaber, Beijing, China).

Cloning of the GCC (ATCCATAAGAGCCGCCACTAAAATAAGACCGATCAA) DNA fragment into pHIS2 was followed by transformation into the Y187 strain. Yeast transformants were screened on an SD (-Trp/-Leu) medium and cultivated on SD (-Trp/-His/-Leu) plates with 100 mM of 3-amino-1,2,4-triazole (3-AT) at 28 °C for 3 d. The corresponding primer sequences are shown in Supplementary Table S1.

### 2.10. Statistical Analysis

Mean values and standard errors were analyzed using Microsoft Excel 2019. Significant differences between controls and treatments, or between time points, were determined with Student’s *t*-test in SPSS 22. GraphPad Prism 8.0 was applied for figure generation.

## 3. Results

### 3.1. Isolation and Sequence Analysis of TaERF109

**Figure 1:**
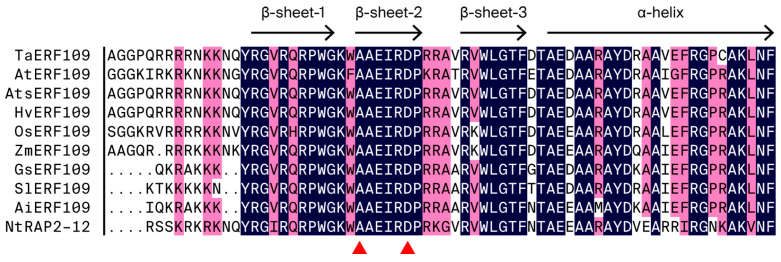
Comparative amino acid alignment of TaERF109 with orthologous sequences across plant species. Alignments were produced in DNAMAN software. The YRG and RAYD elements are indicated below the alignment. The conserved alanine and aspartic acid residues in the AP2 domain are marked with red triangles. AtERF109 is derived from *Arabidopsis thaliana*, AtsERF109 is derived from *Aegilops tauschii* subsp., HvERF109 is derived from *Hordeum vulgare*, OsERF109 is derived from *Oryza sativa*, ZmERF109 is derived from *Zea mays*, GsERF109 is derived from *Glycine soja*, SlERF109 is derived from *Solanum lycopersicum*, AiERF109 is derived from *Arachis ipaensis*, and NtRAP2-12 is derived from *Nicotiana tabacum*.

We identified an AP2/ERF transcription factor with stable expression throughout the infection stage, designated as TaERF109 (GenBank: PX904030), following cloning analysis using transcriptome data from four interactions between *Pgt* and wheat. From ‘Fielder’ leaf tissue, *TaERF109*’s full-length cDNA sequence was isolated using RT-PCR. *TaERF109* had one ORF of 756 bp, encoding 251 amino acid residues. A comparison showed that *TaERF109* was highly similar to *ERF109* of the ERF subfamily in many plants, especially in *A. thaliana*, so it was named *TaERF109* (Figure 1). We found that *TaERF109* has a typical AP2 domain, and primary structure evaluation revealed that the AP2 domain carries two conserved motifs: YPG and RAYD. *TaERF109* was categorized into the ERF subfamily, as its conserved AP2 domain carried alanine (position 14) and aspartic acid (position 19) (Figure 1, Figure S1).

**Figure 2:**
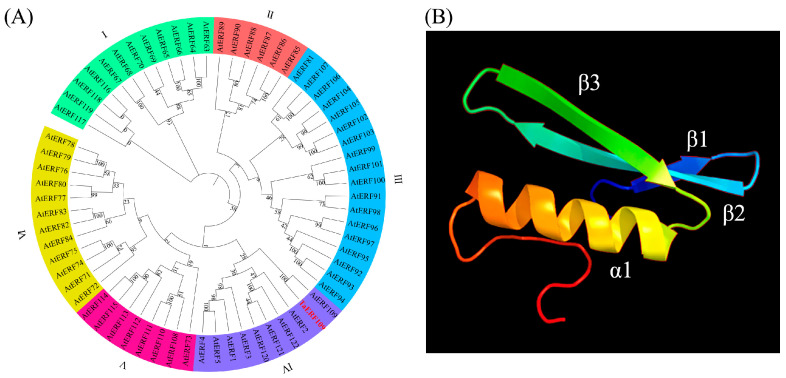
Phylogenetic tree and predicted 3D conformation of TaERF109. (**A**) Tree built using the neighbor-joining method implemented in MEGA 7.0. (**B**) Structural prediction of TaERF109 performed through Phyre2 homology modeling.

Online software analysis revealed that the TaERF109’s molecular weight was 27.306 kDa, and a calculated isoelectric point was 5.25. Residues that might be located in a transmembrane helix yielded a score of 0.3025, falling below the threshold value of 18, signifying the absence of any transmembrane domain within the protein. Furthermore, there was no signal peptide in TaERF109, and online cellular localization prediction demonstrated that TaERF109 was localized in the nucleus. Comparison of the TaERF109 protein sequence with the ERF family protein sequence in A. thaliana revealed that the TaERF109 gene is classified under group IV of the ERF subfamily (Figure 2A). The search results from Phyre2 were used to determine the tertiary structure and revealed that the three-dimensional structure of TaERF109 features a long C-terminal α-helix (α) encased within a three-stranded anti-parallel β-sheet (β1–β3) (Figure 2B).

### 3.2. TaERF109 Expression Was Significantly Induced When Challenged with Pgt

**Figure 3:**
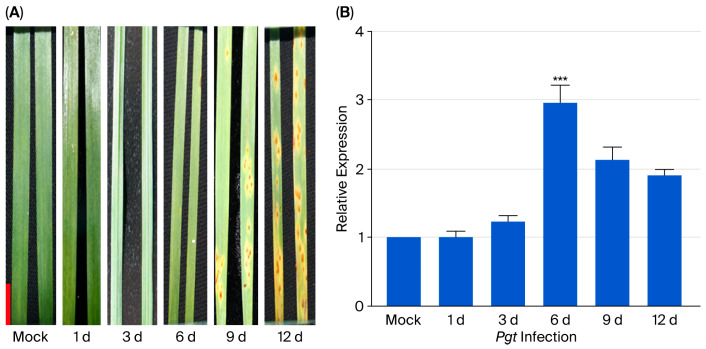
Disease progression and *TaERF109* expression following *Pgt* inoculation. (**A**) Disease symptoms observed on wheat leaves at 1, 3, 6, 9, and 12 dpi. (**B**) Relative transcript level of *TaERF109* in *Pgt*-inoculated leaves at 1, 3, 6, 9, and 12 dpi. Controls were established using samples collected at each time point. TaERF109 expression levels were determined using the 2^−ΔΔCt^ method. Transcript levels were measured with qRT-PCR, normalized to *TaGAPDH*, and shown as changes relative to untreated plants. TaERF109 expression at Mock was set as 1. Statistical analysis was performed with Student’s *t*-test. Error bars indicate SE. *** *p* < 0.001. Data were collected from three biological replicates. The bars represent the standard error of the mean.

The transcript level of *TaERF109* was assessed using qRT-PCR throughout the interaction with *Pgt* to clarify its role in infection response. It had low levels in normal leaves not challenged with *Pgt*, whereas it was up-regulated at 1 and 3 dpi relative to control levels. Its transcript level culminated at 6 dpi, attaining a threefold increase relative to the control. At 9 dpi, expression subsided but remained significantly elevated above baseline levels. Therefore, we speculated that *TaERF109* is involved in the wheat response to *Pgt* (Figure 3).

### 3.3. TaERF109 Expression Was Elicited by Exogenous Ethylene

**Figure 4:**
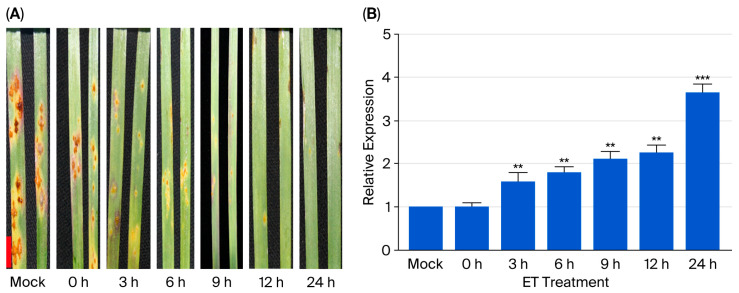
Induction of *TaERF109* expression by exogenous ethylene. (**A**) Resistance phenotype of wheat leaves subjected to ethylene treatment followed by *Pgt* challenge. (**B**) Relative transcript abundance of *TaERF109* in wheat leaves at different time points after exogenous ethylene spraying. Controls were established using samples collected at each time point. *TaERF109* expression levels were determined using the 2^−ΔΔCt^ method. Transcript levels were measured with qRT-PCR, normalized to *TaGAPDH*, and shown as changes relative to untreated plants. *TaERF109* expression at Mock was set as 1. Statistical analysis was performed with Student’s *t*-test. Error bars indicate SE. ** *p* < 0.01; *** *p* < 0.001. Data were collected from three biological replicates. The bars represent the standard error of the mean.

*TaERF109* expression was elicited by ethylene, as revealed via qRT-PCR. *TaERF109*’s transcript level was low under uninfected conditions, but it was significantly elevated 6 h post exogenous ethylene spraying, attaining a 1.8-fold increment relative to the control. After 24 h, the transcript level of *TaERF109* in leaves culminated, attaining a 3.7-fold increment relative to the control. After the ‘Fielder’ leaves sprayed with exogenous ethylene were inoculated with *Pgt*, they also displayed the corresponding resistance phenotype. Considering the preceding experimental findings, exogenous ethylene spraying can be associated with increased expression of *TaERF109*, thus enhancing the wheat resistance to stem rust, and *TaERF109* may mediate the host defense response to *Pgt* through the ethylene signaling pathway (Figure 4).

### 3.4. Subcellular Distribution Revealed Nuclear Localization of TaERF109

**Figure 5:**
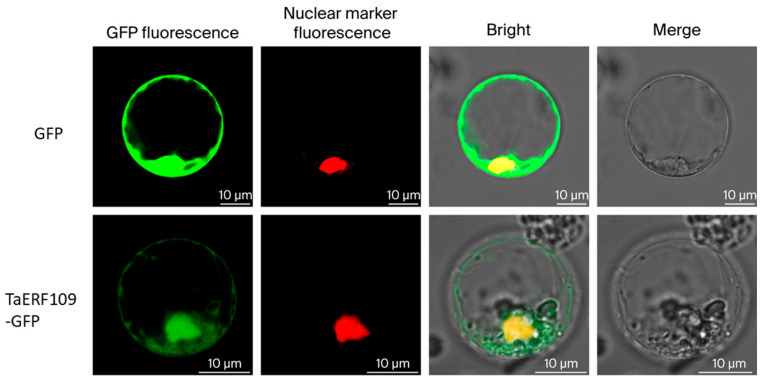
Subcellular localization of the TaERF109 protein. Transient expression of free GFP (control) and TaERF109-GFP under control of the 35S promoter in rice leaf protoplasts. All scale bars are 10 µm.

Expression of the PEGOE35s-BERF109 construct in rice protoplasts was performed to determine TaERF109 localization, confirming its accumulation in the nucleus (Figure 5). It is speculated that the overexpression of the protein leads to the accumulation of a small amount of TaERF109-GFP fusion protein in the cytoplasm. Nuclear localization, a hallmark of transcription factors, was observed for TaERF109, supporting the hypothesis that it functions as a TF.

### 3.5. TaERF109 Silencing Impaired Wheat Resistance to Pgt

**Figure 6:**
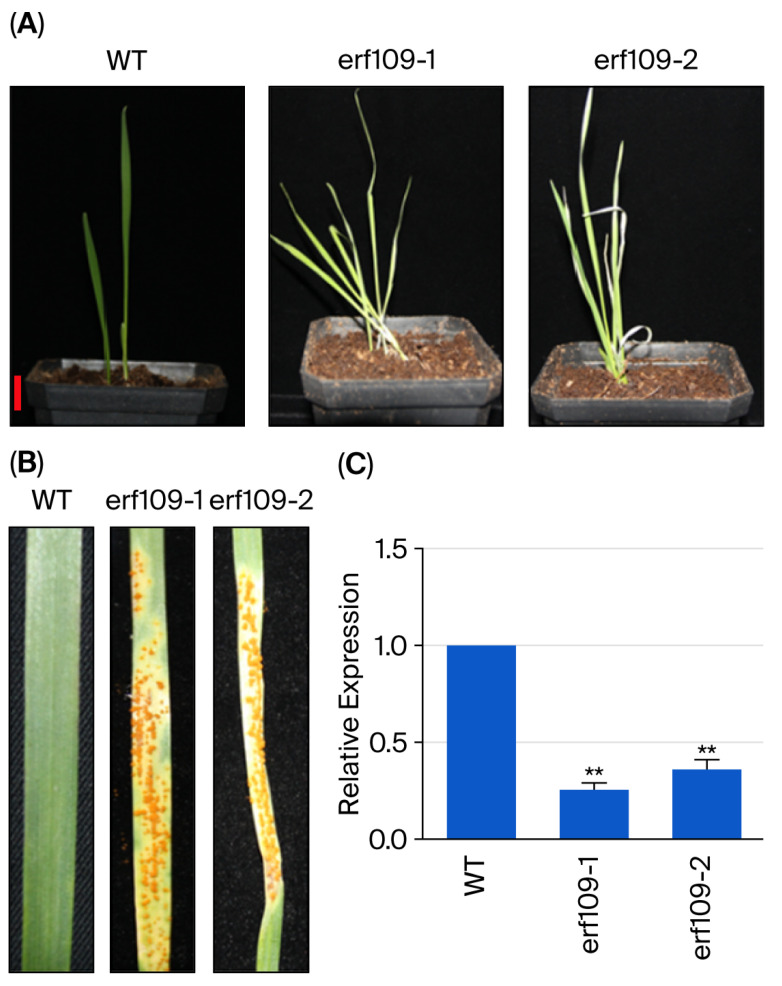
*Taerf109* mutant lines decreased the resistance to *Pgt* (**A**) WT and *Taerf109* mutant lines. (**B**) Disease symptoms on *Taerf109* mutant on detached leaves at 12 dpi. (**C**) Relative *TaERF109* expression in *Taerf109* mutants. *TaERF109* expression levels were determined using the 2^−ΔΔCt^ method. Transcript levels were measured with qRT-PCR, normalized to *TaGAPDH*, and shown as changes relative to untreated plants. *TaERF109* expression at WT was set as 1. Statistical analysis was performed with Student’s *t*-test. Error bars indicate SE.; ** *p* < 0.01. Data were collected from three biological replicates. The experiment was conducted with three independent biological replicates. The bars represent the standard error of the mean.

As previously delineated, the functional engagement of *TaERF109* in the wheat–*Pgt* interaction was analyzed using BSMV-VIGS. Two fragments targeting *TaERF109* in the coding region were designed for silencing, with subsequent amplification employing specific primers. Two-week-old wheat seedlings were inoculated with BSMV: TaPDS, BSMV: *TaERF109*-1, BSMV: *TaERF109*-2, and BSMV: γ at the two-leaf stage. Inoculation with BSMV: TaPDS elicited pronounced photo-bleaching symptoms, whereas BSMV inoculation resulted solely in mild chlorotic mosaic symptoms. Successful *TaERF109* silencing was authenticated in the leaves that underwent inoculation with BSMV: γ, BSMV: *TaERF109*-1, and BSMV: *TaERF109*-2. Thereafter, the fourth leaf of wheat harboring BSMV: γ and *TaERF109*-1/2 was challenged with the *Pgt* isolate 34C3RTGQM. While disease progression proceeded normally across all inoculations, sporulation was marginally elevated in silenced plants relative to BSMV: γ-inoculated plants. To validate the successful silencing of *TaERF109*, its transcript level was assessed via qRT-PCR. Relative to the control, *TaERF109*-silenced plants exhibited markedly reduced transcript accumulation during wheat–*Pgt* interaction, denoting successful *TaERF109* knockdown. These collective observations implied that *TaERF109* may operate as an actuating factor in wheat defense against *Pgt* (Figure 6). Additionally, TaERF109 expression decreased by approximately 0.2-fold and 0.4-fold in Taerf109 mutant lines (Figure 6C). Following *Pgt* inoculation, the Taerf109 mutants demonstrated markedly higher susceptibility compared to 12 dpi (Figure 6B), and lesion sizes in the mutant lines were significantly larger (Figure 6B).

### 3.6. TaERF109 Silencing Affected PR Gene Expression and SOD and POD Activities

**Figure 7:**
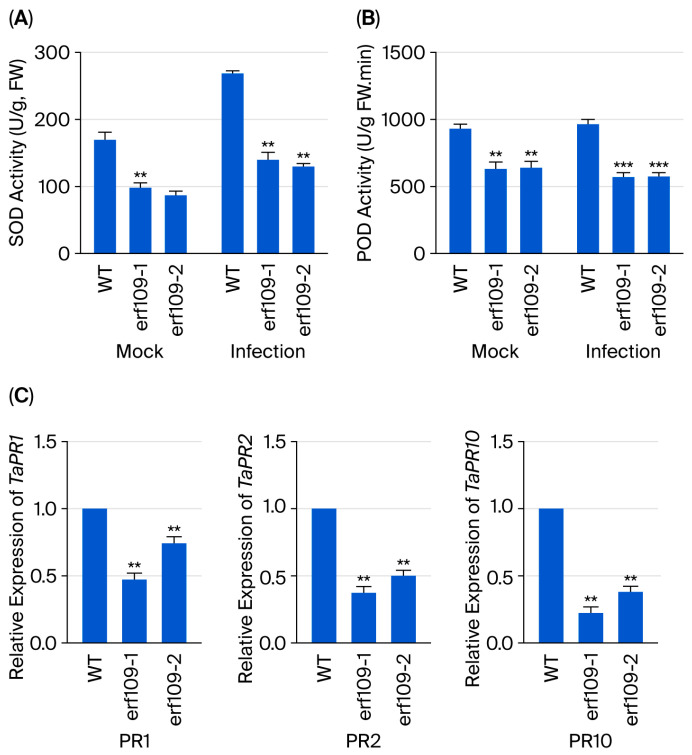
Antioxidant defense and *TaPR* gene expression in *Taerf109* mutant lines under WT and *Pgt* challenge (24 hpi). (**A**,**B**) SOD and POD enzyme activities in *Taerf109* mutant lines. (**C**) Transcript levels of *TaPR1*, *TaPR2*, and *TaPR10* in WT and silenced lines, normalized to Mock-treated WT (set to 1). Data represent the mean ± SE of three biological replicates with technical repeats. TaERF109 expression levels were determined using the 2^−ΔΔCt^ method. Transcript levels were measured with qRT-PCR, normalized to *TaGAPDH*, and shown as changes relative to untreated plants. TaERF109 expression at Mock was set as 1. Statistical analysis was performed with Student’s *t*-test. Error bars indicate SE.; ** *p* < 0.01; *** *p* < 0.001. Data were collected from three biological replicates. The bars represent the standard error of the mean.

SOD and POD are critical antioxidant enzymes involved in the reactive oxygen species (ROS) scavenging process, and they are believed to participate in various defense mechanisms [42]. To examine whether *TaERF109* silencing affects PR gene expression and SOD and POD activities, qRT-PCR detection of *TaPR1*, *TaPR2*, and *TaPR10* transcripts was implemented. Marked attenuation was noted in the transcript abundance of all three genes in *TaERF109*-silenced plants versus the control, signifying a putative role for TaERF109 in the transcriptional up-regulation of specific defense-related genes during wheat–*Pgt* interaction (Figure 7C). TaPR1, TaPR2, and TaPR10 expression and that in Taerf109 mutant lines decreased by approximately 0.5-fold and 0.7-fold, 0.4-fold and 0.5-fold, and 0.2-fold and 0.4-fold (Figure 7C). Concomitantly, SOD and POD activities were substantively diminished in *TaERF109*-silenced plants (Figure 7A,B).

### 3.7. Overexpression of TaERF109 Could Up-Regulate Wheat Resistance to Pgt

**Figure 8:**
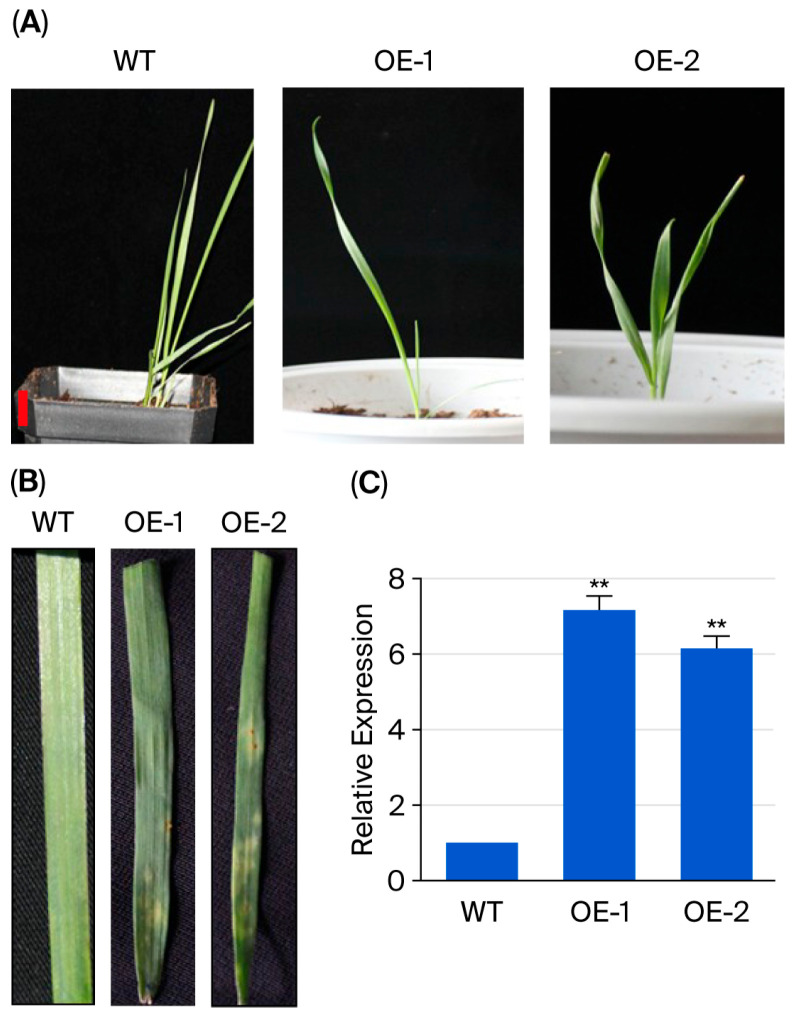
Responses of *TaERF109*-overexpressing wheat to *Pgt*. (**A**) WT and T2 transgenic lines overexpressing *TaERF109*. (**B**) Leaf disease symptoms in T2 lines at 12 dpi. (**C**) *TaERF109*’s relative transcript levels in WT and transgenic plants. Transcript levels were measured with qRT-PCR, normalized to TaGAPDH, and shown as changes relative to untreated plants. TaERF109 expression at Mock was set as 1. Statistical analysis was performed with Student’s *t*-test. Error bars indicate SE.; ** *p* < 0.01. Data were collected from three biological replicates. The bars represent the standard error of the mean.

The positive *TaERF109*-OE seedlings displayed resistance to artificially inoculated 34C3RTGQM (Figure 8). To evaluate the role of TaERF109 in improving resistance to *Pgt* in transgenic wheat, T2 plants featuring two distinct TaERF109 overexpression lines (OE1 and OE2) were analyzed. The expression levels of TaERF109 in these lines were confirmed through qRT-PCR (Figure 8C). Twelve days post-inoculation (dpi), lesion sizes in the overexpressing lines were significantly smaller (Figure 8B), underscoring the beneficial role of TaERF109 in enhancing wheat resistance to *Pgt*.

The effect of TaERF109 overexpression on plants’ resistance to *Pgt* was determined using a leaf inoculation assay. The above results demonstrate that *TaERF109* exerts a positive effect on wheat defense against *Pgt*.

### 3.8. TaERF109 Overexpression Affected PR Gene Expression and SOD and POD Activities

**Figure 9:**
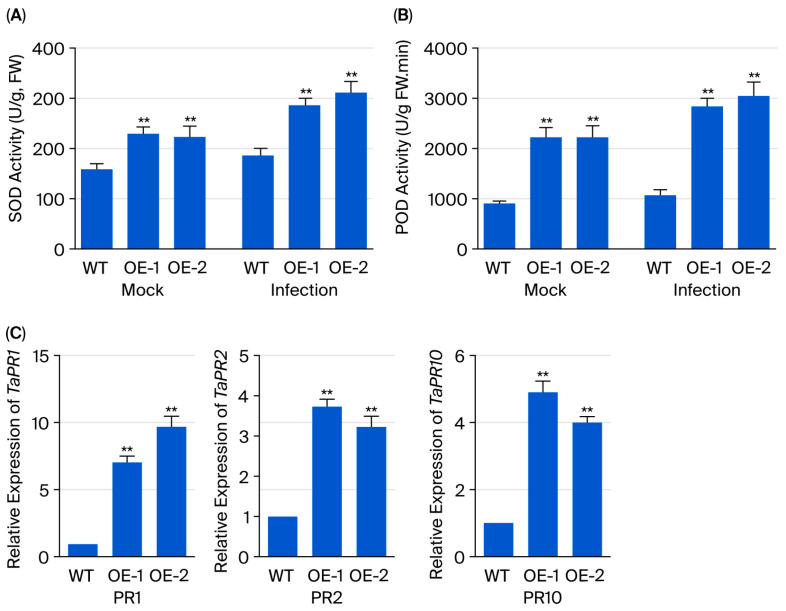
Impact of *TaERF109* overexpression on antioxidant enzyme activity and *TaPR* gene expression in wheat challenged with *Pgt* at 24 hpi. (**A**,**B**) SOD and POD enzyme activities in T2 transgenic lines. (**C**) Relative transcript abundance of *TaPR1*, *TaPR2*, and *TaPR10* in transgenic and WT plants. Values of Mock-treated WT were set as 1. TaERF109 expression levels were determined using the 2^−ΔΔCt^ method. Transcript levels were measured with qRT-PCR, normalized to *TaGAPDH*, and shown as changes relative to untreated plants. *TaERF109* expression at Mock was set as 1. Statistical analysis was performed with Student’s *t*-test. Error bars indicate SE.; ** *p* < 0.01. Data were collected from three biological replicates. The bars represent the standard error of the mean.

Expression profiling of *TaPR1*, *TaPR2*, and *TaPR10* was conducted using qRT-PCR to evaluate the effect of *TaERF109* overexpression. *TaERF109*-OE lines displayed significantly enhanced expression of all three PR genes versus control plants (Figure 9C). TaPR1, TaPR2, and TaPR10 expression and TaERF109 overexpression lines decreased by approximately 7.1-fold and 9.8-fold, 3.7-fold and 3.2-fold, and 4.9-fold and 4.0-fold (Figure 9C). This points to *TaERF109*’s contribution to transcriptional activation of defense-related genes when wheat interacts with *Pgt*. Concomitantly, SOD and POD activities exhibited marked potentiation in *TaERF109*-overexpression lines (Figure 9A,B).

### 3.9. Interaction Between TaERF109 and GCC-Box

**Figure 10:**
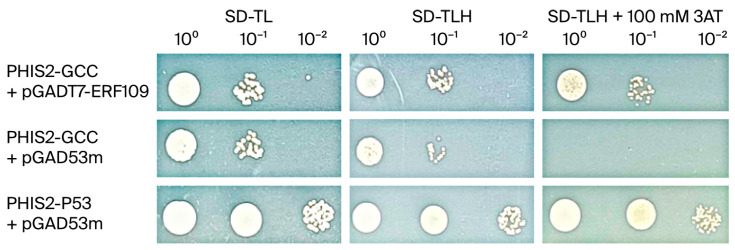
Verification of *TaERF109* binding to GCC-box elements using a Y1H assay. Yeast transformants were plated on SD (-Trp/-Leu) and SD (-Trp/-Leu/-His) media containing 100 mM 3-AT.

ERF genes are known to encode a conserved DNA-binding domain that targets GCC-box sequences, as shown in prior studies [43,44,45]. To further assess the binding specificity of TaERF109 to GCC-box motifs, a Y1H assay was implemented. On an SD (-Leu/-Trp/-His) medium with 100 mM of 3-AT, yeast harboring the WT bait (pHIS2-GCC-box) and prey (pGADT7-TaERF109) vectors grew successfully (Figure 10). These findings prove that TaERF109 exhibits specific binding affinity for GCC-box motifs.

## 4. Discussion

The functional role of AP2/ERF proteins has been substantiated in *A. thaliana* [46,47], rice [48], barley [49], maize [42], tobacco [50], tomato [51], soybean [52], and cotton [53]. Exposure to stress and hormone signals triggers rapid alterations in the activity of ERF TFs. Insights into ERF-regulated mechanisms in plants’ defense against biotic stress are indispensable for improving resistance traits. Contemporary investigations have established that ERF TFs are key regulators in these processes. A rising body of evidence shows that AP2/ERFs are closely involved in defense responses against various pathogens in plants [54].

As typical transcription factors (TFs), AP2/ERFs may function as either transcriptional activators or repressors to regulate plant disease resistance by binding to specific target sequences. For example, transgenic plants expressing AP2/ERFs exhibit enhanced immunity to various pathogens by activating the expression of multiple defense-related genes [55]. For example, *TaERF1* confers multiple stress tolerance [56]. Greater tolerance to *Bipolaris sorokiniana* occurs when TaPIEP1 is overexpressed [57]. ERF TFs operate as both participants in the regulation of abiotic stresses and pivotal players in plant resistance to pathogen invasion. For example, *OsERF922* silencing enhances rice resistance to *Magnaporthe grisea*, while *OsERF922*-overexpression rice is more susceptible to the invasion of *M. grisea* [57]. *Botrytis cinerea* can greatly induce up-regulation of *VaERF* in grape [58]. *VaERF20* overexpression potentiates *A. thaliana* resistance against *Pseudomonas syringae* and *B. cinerea* [59]. ERF1 in *A. thaliana* functions as a pivotal factor downstream of ethylene–jasmonic acid (JA) cross-talk, with its overexpression conferring enhanced plant resistance to multiple necrotrophic fungi [60,61]. A novel wheat ERF TF, TaERF109, was identified and isolated in the present study. The results indicate its implication in pathogen-responsive signaling cascades, demonstrating the capacity to positively modulate wheat resistance to *Pgt*.

Subdivision of the ERF family into ERF and DREB branches relies on the residues found at positions 14 and 19 in the AP2/ERF domain [62]. ERF genes function either as transcriptional activators or repressors, exhibiting binding affinity for GCC-box elements [63]. Most ERF proteins operate as positive regulators of plant immunity [60,64]. Overexpression of *AtERF1*, *AtERF6*, *AtERF96*, and *AtERF104* enhances plant resistance to *B. cinerea* [42]. Analogously, the transcriptional activator *AtERF15* potentiates *A. thaliana* resistance against *Pst* DC3000 and *B. cinerea* [65]. Soybean lines engineered to overexpress *GmERF113* show improved resistance against *Phytophthora sojae* [66]. StERF3-overexpressing plants exhibited decreased expression of defense-related genes and increased susceptibility to *P. infestans*, indicating that StERF3 acts as a suppressor of defense- and stress-related genes in potatoes [67].

Promoter regions of many plant genes tied to disease resistance contain a GCC-box regulatory element. ERFs such as tobacco ERF1/2/3 [68], tomato Pti4/5/6 [69], and rice OsERF83 [70] have been shown to bind to this element. This interaction initiates transcriptional activation of downstream genes implicated in plant defense and osmotic stress responses. ERF overexpression in species such as tobacco and *C. annuum*. GCC-box potentiates both pathogen resistance and abiotic stress tolerance. The GCC-box exhibits responsiveness to multifarious signals, encompassing ethylene, pathogen attack, and mechanical wounding [68]. For instance, *ERF96* binds to GCC-box motifs in promoters of ethylene- and JA-regulated defense genes, thereby enhancing plant resistance to necrotrophic pathogens [45]. Similarly, *ERF1* targets multiple ethylene- and pathogen-responsive gene promoters through GCC-box binding and regulates the expression of secondary ERF genes [68,71]. Overexpression of *ERF1* can partially activate the ethylene response, suggesting that EIN3 also controls other target genes [71].

In the present investigation, TaERF109 protein, similar to other ERF proteins, was substantiated to harbor one conserved AP2 domain incorporating conserved alanine (A) and aspartic acid (D), indicative of its membership in the ERF subfamily. Furthermore, the Y1H assay demonstrated the binding of *TaERF109* to the GCC-box motif, supporting the hypothesis that it regulates defense gene expression at the promoter level. Overexpression of *TaERF109* enhanced wheat resistance to *Pgt*, whereas *ERF109*-mutated plants displayed the opposite phenotype, suggesting that *TaERF109* up-regulates wheat resistance to *Pgt*.

ERF genes are implicated in plant defense response mainly through direct transcriptional modulation of defense-related genes [72]. For instance, AtERF96 potentiates *A. thaliana* resistance to *B. cinerea* by overexpressing *PR3* and *PR4* [45]. *GmERF3* overexpression not only confers potentiated resistance to tobacco mosaic virus (TMV) upon transgenic tobacco but also elicits transcriptional activation of PR1, PR2, and PR4 [73]. Soybean lines carrying *GmERF113* overexpression exhibited stronger resistance to *Pseudomonas*, accompanied by elevated expression of *GmPR1* and *GmPR10-1* [66]. Overexpression of OsERF83 in transgenic rice plants diminished lesion formation following rice blast infection, thereby illustrating the role of OsERF83 in bolstering disease resistance in rice. OsERF83ox plants exhibited increased expression of genes that encode several pathogenesis-related (PR) proteins, including PR1, PR2, PR3, PR5, and PR10 [70]. Overexpression of GbERF regulates the expression of numerous PR and ethylene-responsive genes, crucial for plants’ defense against biotic stress [74]. In the present investigation, *TaPR1*, *TaPR2,* and *TaPR10* exhibited elevated contents in *TaERF109*-overexpression lines following *Pgt* challenge, whereas inverse content patterns were noted in *ERF109*-mutated plants. Therefore, we postulated that TaERF109 may potentiate wheat defense against *Pgt *via direct or indirect modulation of these PR genes.

Production of ROS is an early cellular response after recognizing pathogens, and increased ROS signaling contributes to programmed cell death (PCD) by disrupting metabolism and damaging organelles in plant cells under various stresses [24]. Additionally, POD and SOD, as paramount antioxidant enzymes, facilitate the scavenging of surplus reactive oxygen species in plants, inducing resistance or repairing damage [75]. In the present investigation, SOD and POD activities exhibited a significant augmentation in *TaERF109*-overexpression plants relative to WT but a reduction in *ERF109*-mutated plants, hinting that *TaERF109* potentiates disease resistance. It also shows that *TaERF109* is essential for wheat resistance to *Pgt*. After resistance-related genes are silenced in affinity interaction, fungal biomass will be increased in gene-silenced plant leaves. ERF TFs exert direct controlling effects on PR gene expression [76]. The present investigation revealed attenuated transcript accumulation of *TaPR1*, *TaPR2*, and *TaPR10* in *TaERF109*-silenced plants relative to those in the control, implying TaERF109’s direct or indirect regulatory governance on the expressions of TaPR1, TaPR2, and TaPR10. On the contrary, pronounced up-regulation of these PR genes was noted in *TaERF109*-overexpression plants, further corroborating its aforementioned regulatory governance. Moreover, the transcriptional abundance of *TaERF109* was potentiated through exogenous ethylene application, potentiating disease resistance. We thus postulated that *TaERF109* operates as a positive determinant in wheat resistance to *Pgt*. As shown by the Y1H assay, *TaERF109* mediated the defense pathway through interaction with GCC-box and operated as a modulator potentiating *T. aestivum* L. resistance to *Pgt* in an ethylene-induced manner.

In conclusion, wheat resistance to *Pgt* is enhanced by *TaERF109* overexpression but inhibited by *TaERF109* knockout. Consequently, TaERF109 may represent a promising candidate for augmenting wheat resistance to *Pgt* via genetic engineering. With the swift advancement of modern technologies like next-generation sequencing, multi-omics analysis, the CRISPR/Cas9 gene-editing system, and genome-wide association studies (GWAS), among others, investigating and unraveling the regulatory network of AP2/ERFs will enhance our comprehension of their functions in plant disease resistance.

## 5. Conclusions

We identified *TaERF109* as a novel nucleus-localized AP2/ERF TF in wheat. The ability of TaERF109 to up-regulate wheat resistance to *Pgt* was verified via gene silencing and overexpression assays. According to preliminary functional analysis, TaERF109-induced disease resistance was achieved by increasing defense-related gene expression. These insights establish a theoretical groundwork for subsequent exploration of wheat disease resistance and breeding strategies in the future. The main genes in the ET synthesis pathway are S-adenosylmethionine (SAM), ACC synthase (ACS), and ACC oxidase (ACO). The crucial genes in the ET signaling pathway are ETR (Ethylene receptor), EIN2 (ethylene-insensitive2), EIN3 (ethylene-insensitive3), and EIL (ethylene-insensitive3-like). However, understanding the expression of these genes in this pathway is still incomplete and will be a major research focus in the future.

## Data Availability

The original contributions presented in this study are included in the article/Supplementary Material. The coding sequence of TaERF109 was retrieved from the NCBI database under accession number GenBank: PX904030. Further inquiries can be directed to the corresponding author.

## Supplementary Materials

The following supporting information can be downloaded at https://www.mdpi.com/article/10.3390/pathogens15040387/s1: Table S1: The primer sequences used in this study. Figure S1: Protein sequence alignment of the three homeologues of TaERF109 in wheat. There are three homeologues of TaERF109 in wheat: TaERF109-1A (TraesCS1A01G370600), TaERF109-1B (TraesCS1B01G389700), and TaERF109-1D (TraesCS1D01G376600). Protein sequences were aligned using DNAMAN software.
