# Supplementary material for: TaERF109: A Novel ERF Transcription Factor Contributing to Enhanced Resistance to Puccinia graminis f. sp. tritici Infection in Wheat
Source: Pathogens. 2026 Apr 4;15(4):387. doi: 10.3390/pathogens15040387 (PMC13118380; doi:10.3390/pathogens15040387)
Supplement: Supplementary file 1 [file pathogens-15-00387-s001.zip › pathogens-4100611-supplementary.pdf]

**Table S1.** The primer sequences used in this study

| | | |
|---|---|---|
| Cloning of *TaERF109* | *TaERF109*-F | TCTAGAACTTCAAACAACCATCACCCC |
| | *TaERF109*-R | GAGCTCAAACAAAACTTTTCCATTGATC |
| Overexpression | *TaERF109*-3301-F | ACTAGGGTCTCGCACCCAGCAAGAACACACAGACCTTCA |
| | *TaERF109*—3301-R | ACTAGGGTCTCTCGCCATAGGCATCCAATCGTCTCTGT |
| Yeast one-hybrid | pGADT7- *TaERF109*-F | ATGGAGGCCAGTGAATTCATGAAGGCGAGCCGGGAGTACATGAT |
| | pGADT7- *TaERF109*-R | CTCGAGCTCGATGGATCCCTAATAGGCATCCAATCGTCTCTGT |
| qRT-PCR | *TaGAPDH* -QF | CTGCATCATACGATGACATC |
| | *TaGAPDH* -QR | TGTCACCGACAAAGTCAGTG |
| | *TaERF109*-QF | GAACGGCGGGCAGGAAA |
| | *TaERF109*-QR | AGAGGGCGGGAACCAGA |
| | *TaPR1*-QF | CTGGAGCACGAAGCTGCAG |
| | *TaPR1*-QR | CGAGTGCTGGAGCTTGCAGT |
| | *TaPR2*-QF | CTCGACATCGGTAACGACCAG |
| | *TaPR2*-QR | GCGGCGATGTACTTGATGTTC |
| | *TaPR10*-QF | CGTGGAGGTAAACGATGAG |
| | *TaPR10*-QR | GCTAAGTGTCCGGGGTAAT |
| Subcellular Localization | *TaERF109*-F | CACGGGGGACTCTTGCCACC |
| | *TaERF109*-R | GACACGCTGAACTTGTGG |
| Primer for VIGS | *BSMV-TaERF109-F:* | TTTCTAAGGAAGGGCCGCTGCCTGACGCCACGGT |
| | *BSMV-TaERF109-R:* | TTAACCACCACCACCGGAGCCACCACTGCCGTTC |

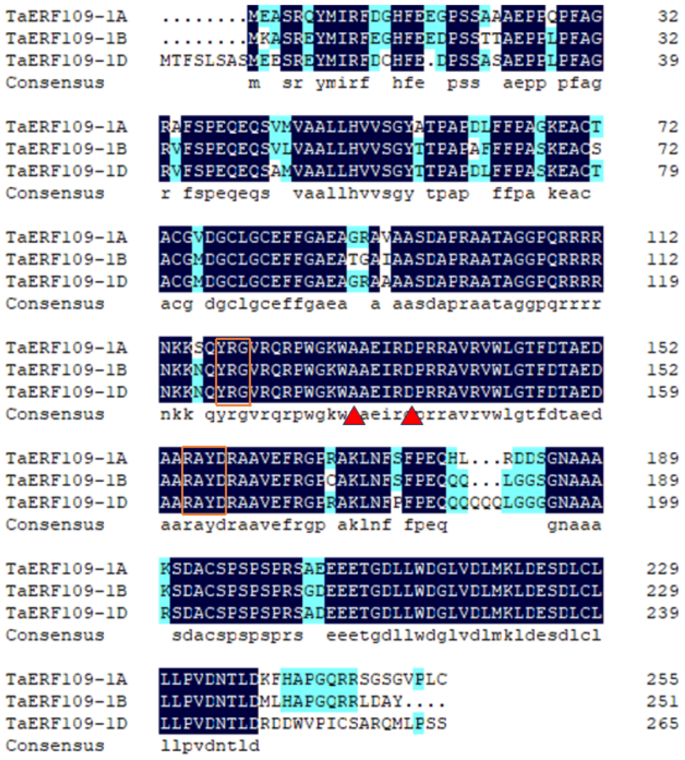


**Figure S1.** Protein sequence alignment of the three homeologues of TaERF109 in wheat. There are three homeologues of TaERF109 in wheat, that is, TaERF109-1A (TraesCS1A02G370600), TaERF109-1B (TraesCS1B02G389800) and TaERF109-1D (TraesCS1D02G376600). Protein sequences were aligned using DNAMAN8 software.
